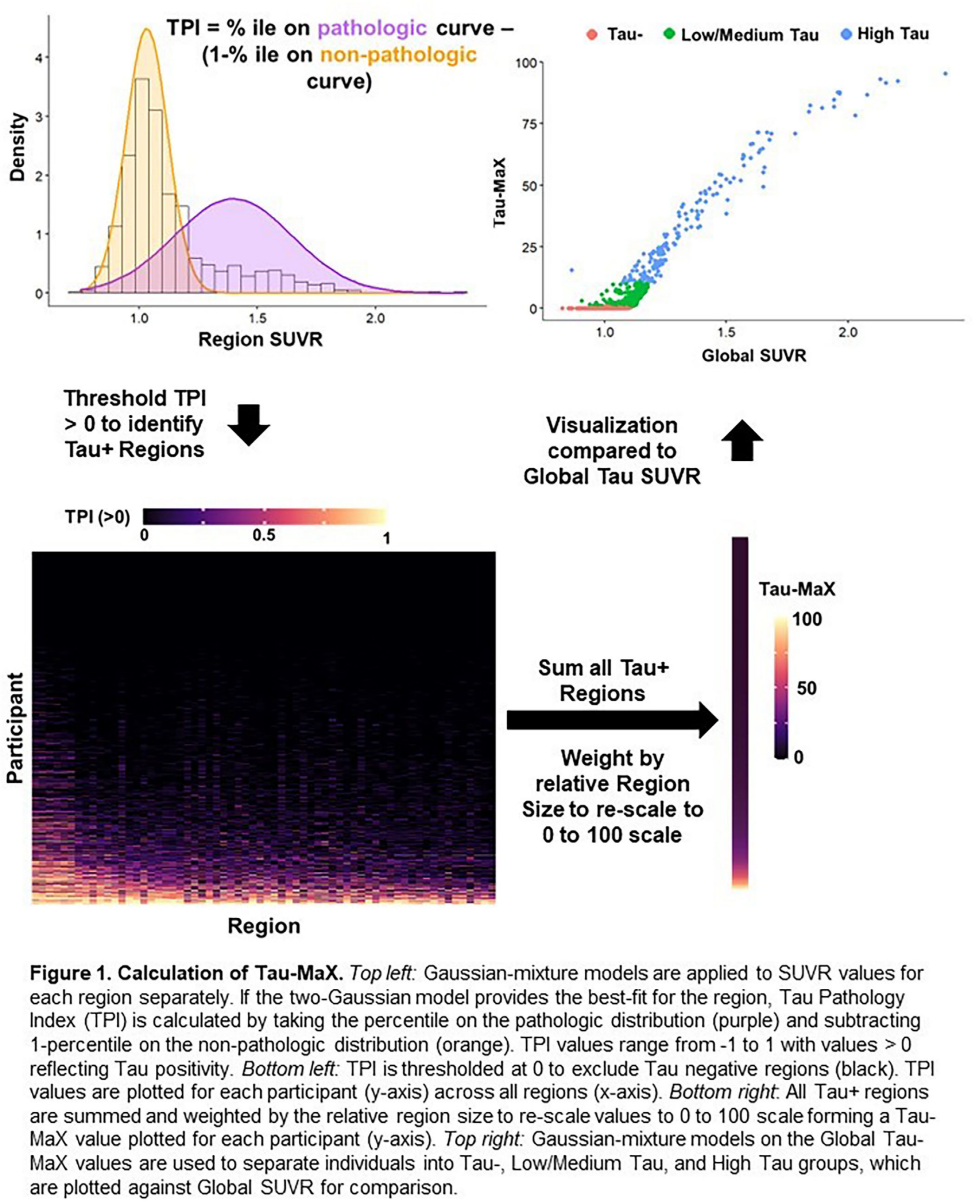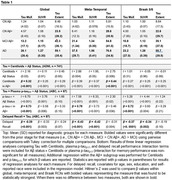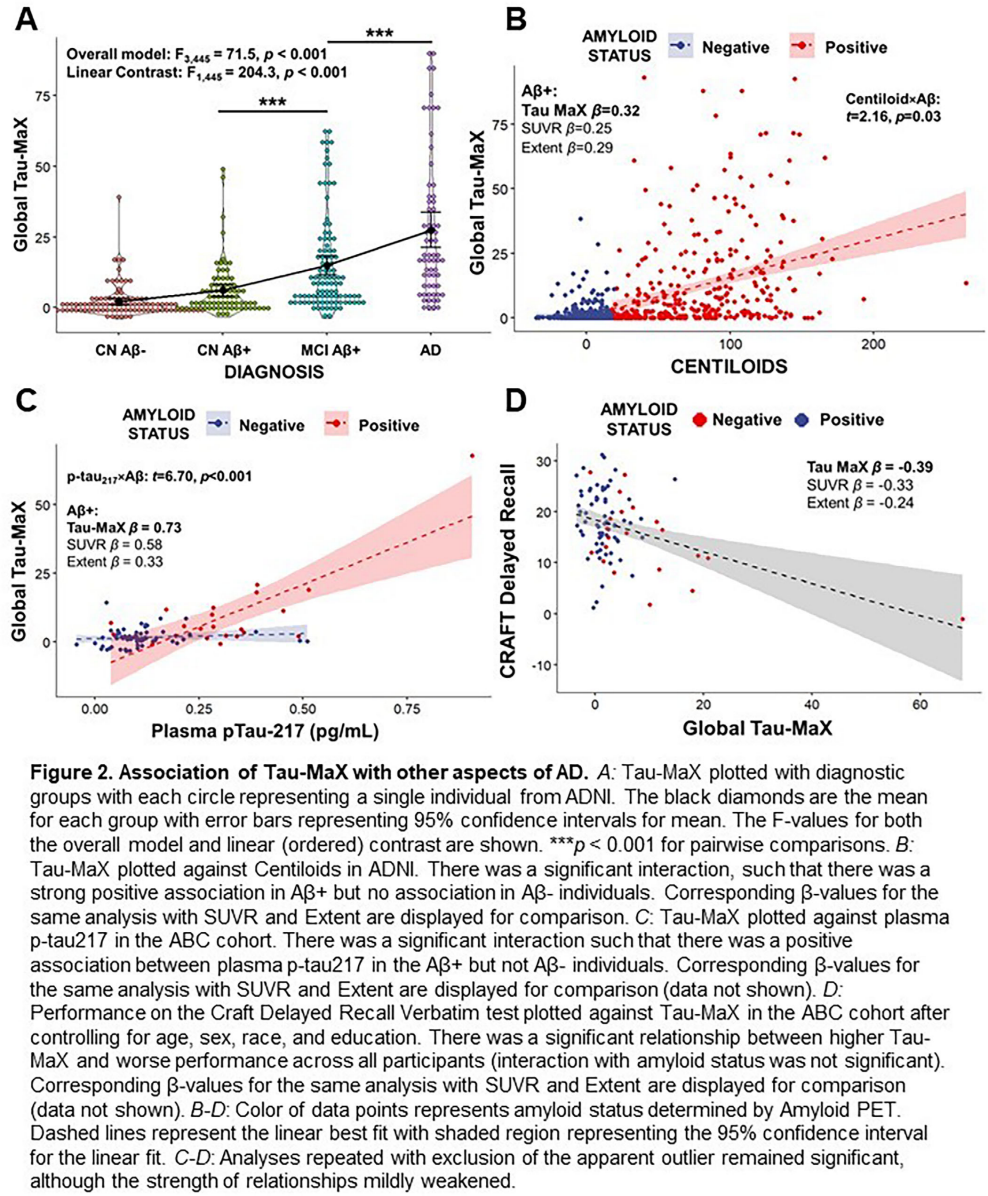# Tau Magnitude and eXent–Tau‐MaX: Capturing tau spread and local accumulation provides a robust metric of tau burden

**DOI:** 10.1002/alz.095756

**Published:** 2025-01-09

**Authors:** Christopher Brown, Sandhitsu R. Das, Katheryn A Q Cousins, Thomas F. Tropea, Alice Chen‐Plotkin, Eddie B Lee, Ilya M. Nasrallah, John A. Detre, Paul A. Yushkevich, Leslie M. Shaw, David A Wolk

**Affiliations:** ^1^ University of Pennsylvania, Philadelphia, PA USA

## Abstract

**Background:**

Measures of tau burden have typically relied upon measures of magnitude, such as mean standardized uptake value ratio (SUVR), or extent, such as number of tau positive regions. However, heterogenous patterns of tau spread and accumulation present challenges to using these measures in isolation to quantify tau burden. Therefore, we hypothesized that a combined measure of tau magnitude and extent (Tau‐MaX) would outperform either measure in isolation.

**Method:**

Baseline ^18^F‐flortaucipir PET scans from 916 participants from ADNI (n = 741) and Penn Aging Brain Cohort (ABC, n = 175) were used for this study. SUVRs, using an inferior cerebellar reference region, were converted to Tau Pathology Indices (TPI) based on mixed‐Gaussian models (Figure 1). Regions with TPI > 0 (reflecting tau positivity) were weighted by region size and summed to form a measure of Tau Magnitude and eXtent (Tau‐MaX, Figure 1). This process was carried out globally and within two common meta‐ROIs: meta‐temporal and Braak 5/6. For each of these three meta‐ROIs, we compared diagnostic group differences, association with Centiloid values from amyloid PET, correlation with Fujirebio Lumipulse p‐tau_217_ measures, and delayed recall performance using Tau‐MaX, mean SUVR, or extent of tau positivity. Comparison between associations was performed using Hittner’s Z.

**Results:**

Group differences and results from regression analyses are shown in Table 1. There was a stepwise increase in all measures across diagnostic groups, although extent measures were more sensitive for detecting differences between CN Aβ+ and CN Aβ‐ individuals. Similarly, extent measures were more closely associated with Centiloid values in Aβ+ individuals in the meta‐temporal and Braak ROIs, but Tau‐MaX was more sensitive globally. Tau‐MaX had a stronger relationship with plasma p‐tau_217_ compared to either SUVR or extent. Finally, Global Tau‐Max and meta‐temporal Tau‐Max and SUVR were the best predictors of delayed recall performance.

**Conclusion:**

Tau‐MaX provides a robust metric of tau burden by incorporating both magnitude and extent. Further, the region agnostic approach may be particularly useful in more heterogenous cohorts that do not follow canonical Braak patterns of spread and accumulation. Longitudinal analyses of these measures will help identify their utility as markers of therapeutic response.